# Resistance to visceral leishmaniasis is severely compromised in mice deficient of bradykinin B2-receptors

**DOI:** 10.1186/1756-3305-5-261

**Published:** 2012-11-14

**Authors:** Dirlei Nico, Daniel Ferreira Feijó, Naiara Maran, Alexandre Morrot, Julio Scharfstein, Marcos Palatnik, Clarisa Beatriz Palatnik-de-Sousa

**Affiliations:** 1 Instituto de Microbiologia Paulo de Góes, CCS, Universidade Federal do Rio de Janeiro (UFRJ), Avda. Carlos Chagas 373. Cidade Universitária, Ilha do Fundão, Rio de Janeiro, Caixa Postal 68040, 21941-902, Brazil; 2 Instituto de Biofísica Carlos Chagas Filho, Universidade Federal do Rio de Janeiro (UFRJ), Rio de Janeiro, 21949-900, Brazil; 3 Hospital Universitário Clementino Fraga Filho-Faculdade de Medicina, Universidade Federal do Rio de Janeiro (UFRJ), Rio de Janeiro, CEP 21941-913, Brazil

**Keywords:** B2 kinin receptor, Visceral leishmaniasis, Leishmania (L.) donovani, Leishmania (L.) chagasi, Susceptibility, Resistance

## Abstract

**Background:**

Kinins liberated from plasma–borne kininogens, are potent innate stimulatory signals. We evaluated whether resistance to infection by *Leishmania (L.) chagasi* depends on activation of G-protein coupled bradykinin B2 receptors (B2R).

**Findings:**

B2R ^−/−^ C57BL/6 knock-out (KOB2) and B2R^+/+^ C57BL/6-wild type control mice (C57) were infected with amastigotes of *Leishmania (L.) chagasi*. Thirty days after infection, the KOB2 mice showed 14% and 32% relative increases of liver (p< 0.017) and spleen weights (p<0.050), respectively, whereas liver parasite load increased 65% (p< 0.011) in relation to wild type mice. The relative weight increases of liver and spleen and the parasite load were positively correlated (R = 0.6911; p< 0.007 to R = 0.7629; p< 0.001, respectively). Conversely, we found a negative correlation between the increased liver relative weight and the weakened DTH response (a strong correlate to protection or natural resistance to VL) or the decreased levels of IgG2b antibodies to leishmanial antigen. Finally, we also found that IFN-γ secretion by splenocytes, an adaptive response that was significantly decreased in KOB2 mice (p< 0.002), was (i) negatively correlated to the increase in liver LDU (R = −0.6684; p = 0.035) and liver/body relative weight (R = −0.6946; p = 0.026) and (ii) positively correlated to serum IgG2b levels (R = 0.8817; p = 0.001).

**Conclusions:**

We found that mice lacking B2R display increased susceptibility to the infection by *Leishmania (L.) chagasi.* Our findings suggest that activation of the bradykinin/B2R pathway contributes to development of host resistance to visceral leishmaniasis.

## Findings

VL is a chronic and vector-borne potentially fatal parasitic disease caused by the *Leishmania (L.) donovani* / *Leishmania (L.) infantum / Leishmania (L.) chagasi* complex which is associated to fever, malaise, anaemia, caquexia, fatigue, enlargement of the liver, spleen and lymph nodes, hypergammaglobulinemia and to the progressive suppression of cellular immunity [1,2]. Anti-parasite immune response against the parasite is still present at early infection and recovery after cure subsequent to chemotherapy; this can easily been assessed by the determination of the delayed type hypersensitivity following injection of a leishmanial antigen [3-5]. The variable degrees of susceptibility or natural resistance to VL have been the focus of intense studies in the mice and dog models [6,7] and in humans [8] since the 70’s. A genetic basis for the susceptibility to VL was described in mice, dogs and humans [7,9-11]. It is well known that innate immunity plays a pivotal role in host resistance to VL. There is now awareness that alert signals expressed by pathogens and/or generated by injured tissues might link the innate system to adaptive immunity. Tissue injury can lead to bradykinin or Lysyl-bradykinin excision from high and low molecular weight kininogens by the respective action of the serine proteases, plasma and tissue kallikrein [12]. Once liberated, the short-lived kinins induce inflammatory responses (e.g. increased blood flow, oedema formation, vasodilatation and pain sensation) through the activation of two distinct subtypes of G-protein coupled bradykinin receptors (BR). One of these, B_2_R, is constitutively expressed by a broad range of host cell types, e.g., endothelial cells, epithelial cells neurons and dendritic cells (DCs) [12,13]. In mice infected subcutaneously by *Trypanosoma cruzi*, immature CD11c^+^ DCs detect the presence of kinin peptides liberated in peripheral sites of infection through the signalling of B_2_R [14]. Upon maturation, the antigen-bearing B_2_R^+/+^ DCs emigrate to secondary lymphoid tissues of chagasic mice where they prime naive T cells while steering Th1 polarization via the IL-12-dependent pathway [13-15]. Using a systemic model of *T.cruzi* infection, Monteiro et al. showed that B_2_R^−/−^ mice succumb to acute parasite challenge [13]. After showing that B_2_R^−/−^ chagasic mice failed to optimally develop type-1 T cell effectors, these authors showed evidence that their susceptible phenotype is a consequence of impaired maturation of splenic B_2_R^−/−^ DCs [13]. Interestingly, in Balb/c infected (mucosally) with the periodontal bacteria *Porphyromonas gingivalis*, the activation of the kinin/B2R pathway generated IL-17 and IFN-γ- producing T cells whereas infected C57BL/6 mice exclusively generated IFN-γ-producing T cells, (likewise in B2R-dependent manner) [16].

Regarding VL, *in vivo* studies in mice and hamsters showed that *Leishmania (L.) donovani* and *Leishmania (L.) chagasi* promastigotes evoke inflammatory oedema through the proteolytic release of kinins [17]. In addition, *in vitro* studies showed that activation of B_2_R enhance parasite uptake by splenic adherent cells while reducing amastigote outgrowth in inflammatory macrophages [17].

The Balb/c strain is extremely susceptible to tegumentary leishmaniasis infections by *L. (L.) mexicana*, *L. (L.) major*[18-21] and *L. (L.) braziliensis*[19] and the C57BL/6 strain is resistant to the infection by *L. (L.) major*[19-21] and *L. (L.) braziliensis*[19]. In the case of VL, on the other hand, the C57BL/6 strain was considered equally [18-20,22,23] or more susceptible [18] than the Balb/c strain, to the infections by *L.(L.) donovani*, *L.(L.) infantum* or *L.(L.) chagasi*, both developing high rates of parasites loads in liver during early acute infection. Furthermore, reflecting the genetic variability in the H2 locus, the Balb H-2 ^d/d^ haplotype is associated with persisting visceral leishmaniasis infection and the H-2^b/b^ haplotype with substantial recovery by day 130 after infection [18]. However, in spite of the impressive number of reports of VL studies performed in the Balb/c model [5,19-22], all the transgenic knock-out mice were developed within the C57BL/6 background and there are no B2R knock-out mice of Balb/c genetic background.

In this investigation we evaluated the potential role of the bradykinin receptor B2R in resistance to VL using the C57BL/6 wild type mice and its BR2 knock-out mutant (C57BL/6/BR2^−/−^) which shares the same C57BL/6 genetic background.

## Methods

C57Bl/6 B_2_R^−/−^ knock-out (KOB2) [24] and C57BL/6- B2R^+/+^ wild type control mice (C57) originated from breeding colonies kindly donated by Dr J.B. Pesquero (UNIFESP, São Paulo, Brazil) were maintained in our animal facilities (LAT- IBCCF, UFRJ). Deletion of the entire coding sequence of kinin B2 receptors was achieved according to the methodology previously described by Rupniak *et al*. [25]. Briefly, the generation of transgenic B2R mice was achieved by transfection of embryonic stem cells from J129 mice with a targeting vector designed to disrupt the B2 receptor gene. Then hybrid mice were obtained of the J129 and the C57 strain taking advantage the fertility capabilities of the C57 strain. Once the hybrid is obtained it is possible to generate a pure C57BL/6 B2R ^−/−^ strain through repeated back-crossing with C57BL/6 [25]. All mouse studies followed the guidelines set by the National Institutes of Health, USA and the Institutional Animal Care and Use Committee approved the animal protocols (IBCCF, UFRJ, protocol IMPPG-007).

Female C57BL/6 BR^+/+^ and B_2_R^−/−^ mice, 8-week-old, were challenged intravenously with 3×10^7^*L. (L.) chagasi* amastigotes obtained from infected hamsters spleens. The strain used for challenge (IOC-L 3324) was originally isolated from the spleen of an infected dog of Andradina, São Paulo, Brazil and taxonomically characterized as *Leishmania (L.) chagasi* by the CLIOC-WDCM 731 (Instituto Oswaldo Cruz *Leishmania* collection, Rio de Janeiro, Brazil). Thirty days after infection, mice were euthanized using gaseous Carbon Dioxide and the liver parasite load was evaluated in Giemsa-stained smears and expressed in LDU values (Leishman Donovan units of Stauber = number of amastigotes per 1000 liver cell nuclei/mg of liver weight) [6,18]. The increases in liver and spleen/corporal relative weight were also recorded as clinical signs of VL.

The DTH against *L. (L.) donovani* lysate was measured in the footpads on day 28 after infection, as described earlier [26]. Briefly, mice were injected intradermally, in the right front footpad, with 10^7^ freeze-thawed stationary phase *Leishmania (L.) donovani* (LD 1S/MHOM/SD/00-strain 1S) promastigotes in 0.1 ml sterile saline solution. The contra-lateral footpad received 0.1 ml saline, as control. Footpad thicknesses were measured with a Mitutoyo apparatus, at 0, 24 and 48 h after injection. At each measurement, the values of the saline control were subtracted from the reaction due to the *Leishmania* antigen. Previous experiments performed in mice and CB hamsters demonstrated that the saline treated footpads returned to base levels 24 h after inoculation [26].

Serum antibody responses were monitored by an enzyme-linked immunosorbent assay (ELISA) using as antigen the recombinant Nucleoside hydrolase (NH36) of *Leishmania (L.) donovani*[27] or the freeze and thawed lysate of stationary phase promastigotes of *Leishmania (L.) chagasi* (LIOC 579). The NH36 protein cloned into the pET28b expression system was expressed in *E. coli* Bl21DE3 cells and purified in a Ni-NTA column (Qiagen) [27]. Thirty days after infection with *L. (L.) chagasi*, sera were collected and the ELISA assay performed by using 2 μg of recombinant NH36 or promastigote lysate per well and goat anti-mouse IgG1 and IgG2b-horseradish peroxidase conjugate (Southern Biotechnology Associates, Birmingham, Ala.) at 1:1,000 dilution in blocking buffer. The reaction was developed with *o*-phenylenediamine (Sigma), interrupted with 1 N sulphuric acid, and read at 492 ηm. Sera were analyzed at a 1/100 dilution in triplicate. Positive and negative control sera were included in each test.

Furthermore, for the assay of IFN-γ secretion we used 10^6^ splenocytes after 5 days of *in vitro* culturing at 37°C and 5% CO2 in RPMI medium [27] and/or 10^6^ freeze-thawed stationary phase *Leishmania (L.) chagasi* (IOC L579) promastigotes in sterile saline solution obtained from infected and uninfected C57 and BOK2 mice. Secretions of IFN-γ were evaluated in the supernatants with an enzyme-linked immunosorbent assay (ELISA) using purified antimouse IFN-γ (clone R4–6A2; BD Bioscience), biotin-conjugated antimouse IFN-γ (clone XMG1.2; BD Bioscience), streptavidin-alkaline phosphatase (BD Bioscience) and developed with ELISA Development Kit from R&D System according to the manufacturer’s instructions.

### Statistical analysis

Means of variables were compared by Kruskall Wallis and Mann Whitney non-parametrical tests (Analyze-it). Correlation coefficient analysis was determined on a Pearson bivariate, two tailed test of significance (SPSS for windows). The values of R^2^ , which represents the fraction of the total variance in Y that can be explained by the variation in X, were obtained using linear regression analysis (Analyze-it).

## Results

**Figure 1:**
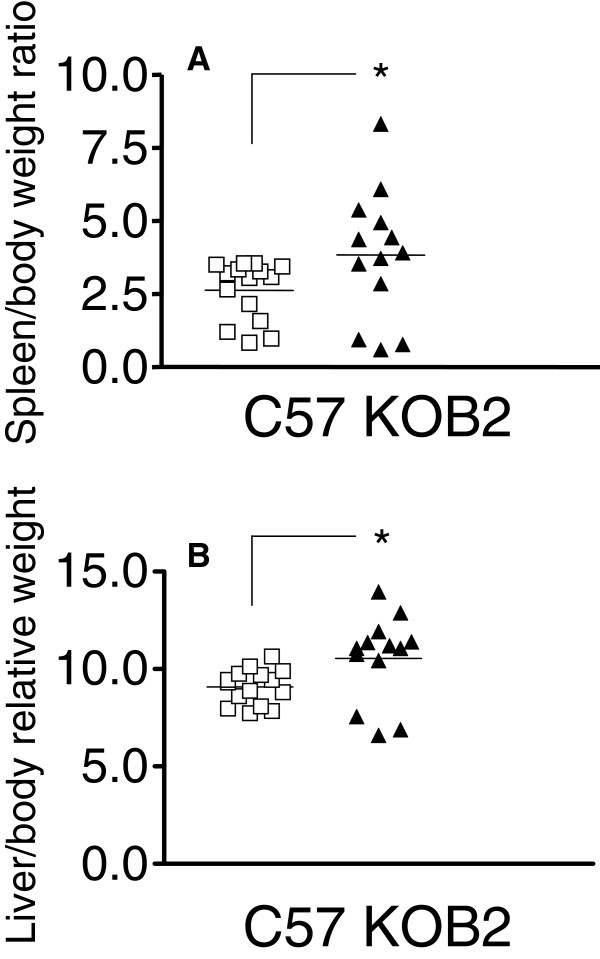
**Spleen and liver/body relative weights are increased in B**_**2**_**R-deficient mice infected with *L. (L.) chagasi.*** The body weight was measured in grams at day 30 and the spleen (**A**) and liver / body relative weights (grams of organ weight x 100 / grams of body weight) (**B**) were determined in B_2_R^+/+^ (C57) and B_2_R^−/−^ mice (KOB2). Data represent the individual results for each group of mice (n = 3–5) of three independent experiments. Asterisks indicate significant differences between groups as disclosed by the Mann Whitney analysis.

In order to evaluate whether kinins may contribute to immune resistance to VL infection, wild type (C57) [22,23] and B_2_R^−/−^ (KOB2) [13] mice were infected intravenously with *Leishmania (L.) chagasi* amastigotes. All animals were euthanized 30 days after infection in order to ascertain the clinical parameters that characterize advanced VL, e.g., by measuring liver and spleen/body relative weight, both of which are strong indicators of advanced VL [22,27]. Data shown in Figure 1 disclosed that the spleen (p = 0.050) (Figure 1A) and liver/body relative weight (Figure 1B) (p = 0.017) were significantly increased in KOB2-infected mice as compared to the C57 mice. These results suggest that B_2_R-receptor is implicated in the development of natural host resistance to experimental VL.

**Figure 2:**
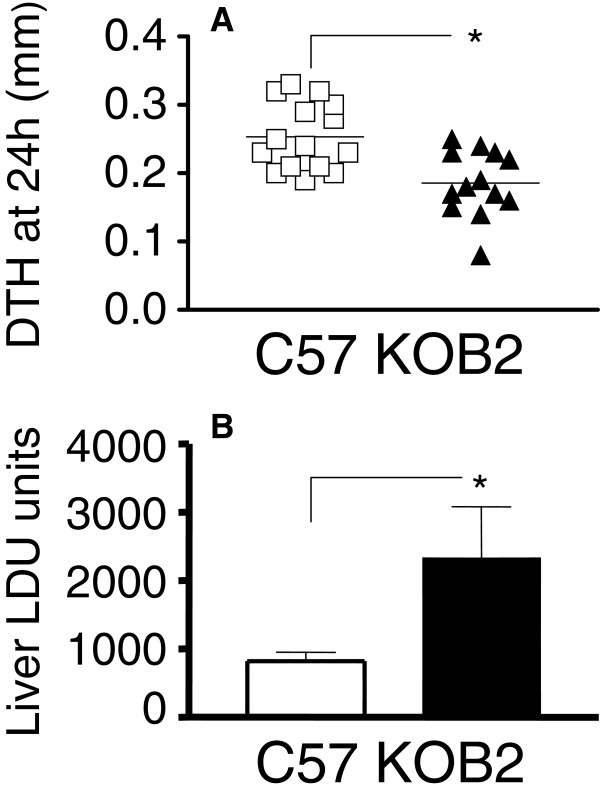
**Increased parasite tissue burden and impaired DTH response in B**_**2**_**R deficient mice. **(**A**) The *y*-axis represents the individual results of the thickness of skin reaction in mm at 24 h after intradermal injection of 10^7^ freeze–thawed stationary phase promastigotes, as measured on day 28 after infection in B_2_R^+/+^ (C57) and B_2_R^−/−^ mice (KOB2)*.* The values of the contra-lateral saline control were subtracted from the reaction due to *Leishmania* antigen. Data represent the results of three independent experiments performed with 3–5 mice for each treatment. (**B**) The *y*-axis represents the average liver parasitic load in Leishman-Donovan units of Stauber (LDU= number of amastigotes / 1000 cell nuclei × organ weight in mg) of two independent experiments (5 mice/treatment), at 30 days after infection with 3 x 10 ^7^ amastigotes of *Leishmania (L.) chagasi.* T bars represent the SE values. Asterisks indicate significant differences between groups as disclosed by the Mann Whitney analysis.

Next, we measured DTH responses to the promastigote leishmanial antigen to compare the efficiency of cellular immunity in mice infected with *Leishmania (L.) chagasi*[27]. Our data showed that the DTH response was significantly lower in the KOB2 mice as compared to response of the wild type infected animals (Figure 2A) (p = 0.003). These results suggest that the cellular immunity is impaired in mice lacking B_2_R, thus raising the possibility that activation of the kinin/B_2_R pathway is required for optimal development of host resistance to LV. Consistent with this, KOB2 mice displayed an increase in the relative weight of spleen and liver (Figure 1 A and B), whereas the DTH responses, as predicted, were impaired (Figure 2A). Noteworthy, the parasite load was significantly increased in the liver of the mutant mice (65%; p = 0.011; mean ± SE = 2327.64 ± 795.41) over values of wild type mice (818.73 ± 139.55) (Figure 2B).

Although we have not characterized the mechanisms underlying the immune dysfunction of B_2_R^−/−^ mice infected with *Leishmania (L.) chagasi,* studies in *T. cruzi* infected B2R-deficient mice, which also exhibited a susceptible phenotype, revealed that host resistance to systemic infection was impaired as a result of deficient DC responsiveness to endogenously released kinins [13,15].

**Figure 3:**
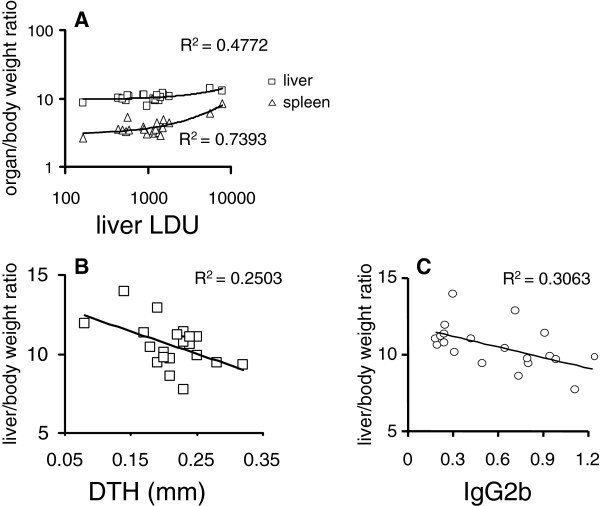
**Correlation between the clinical signs of visceral leishmaniasis and the cellular versus antibody responses to leishmanial antigen. **Data represented in bivariate graphics: (**A**) the positive correlation between the individual increase of spleen and liver relative weights and the increase in liver parasite load; and (**B**) the negative correlation between the increase in liver relative weight and the decrease in DTH response to leishmanial antigen, and (**C**) the increase in liver relative weight and the decrease in anti-NH36 IgG2b antibody response. The results were expressed on graphs as scattering of individual values. R-squared (R^2^) (coefficients of determination) estimates are shown on graphs. Trend lines were added.

Our study showed that *Leishmania (L.) chagasi* infection of B_2_R^−/−^ mice accurately reproduced the advanced VL clinical signs. For example, we found a highly significant positive correlation between the spleen/body (R = 0.8600; p < 0.001), liver/ body relative weights (R = 0.6911; p < 0.007) and liver parasite load, all of which were increased (Figure 3A). Likewise, the increases in liver and spleen/body relative weights were also positively correlated (R = 0.7629; p < 0.001). Furthermore, the decrease in DTH response against leishmanial antigen was negatively correlated to the increase in liver/ body relative weight (R = −0.5003; p = 0.025) (Figure 3B). Since DTH is a strong correlate of protection and required for optimal development of resistance to VL [27-30], we may infer that the impaired DTH observed in KOB2 mice is an expression of deficient cellular immunity.

**Figure 4:**
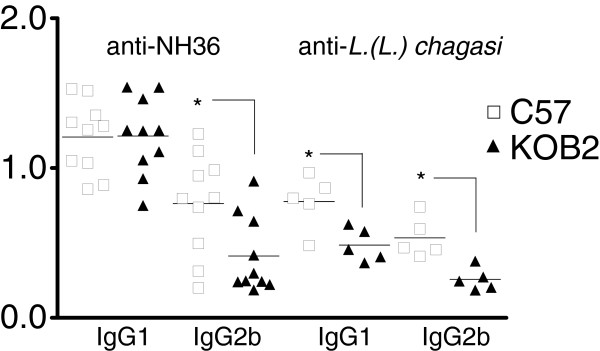
**Evaluation of the IgG1 and IgG2b response in sera of mice deficient of B**_**2**_**R. **ELISA assay showing anti-Nucleoside hydrolase (NH36) and anti-*L.(L.) chagasi* promastigote lysate IgG1 and IgG2b antibodies in B_2_R^+/+^ wild type (C57) and B_2_R^−/−^ mice (KOB2) intravenously infected with 3 x 10^7^ amastigotes of *Leishmania. (L.) chagasi.* Results are shown as the individual absorbency values of 1/100 diluted sera obtained from two (NH36) or one (lysate) experiment with 5 mice per treatment in each experiment, at day 30 after infection. Asterisks indicate significant differences between groups as disclosed by the Mann Whitney analysis.

As an adjuvant to conclude on the immunological status of the animals we also evaluated the anti-IgG1 and IgG2b antibodies in mouse sera. Our ELISA assays revealed that levels of IgG2b anti-NH36 antibodies in the infected C57 mice were significantly higher (p = 0.018) as compared to the KOB2 mice (Figure 4). The increase in IgG2b anti-NH36 antibodies and in DTH response were positively correlated (R = 0.4587; p = 0.042). Furthermore, similar to the DTH response (Figure 3B), the IgG2b increase was also negatively correlated to the increase in liver / body relative weight (R = −0.5536; p = 0.011) (Figure 3C). When the ELISA assay was performed, using as antigen the total *L. (L.) chagasi* lysate, the levels of IgG1 and IgG2b antibodies in the infected C57 mice were significantly higher (p = 0.008 and p= 0.032, respectively) as compared to the KOB2 mice (Figure 4). The IgG1 or IgG2b antibodies themselves, on the other hand, showed no significant differences in their reactivity to NH36 or lysate, either for C57 or BOK2 mice. Furthermore, the anti-lysate IgG1 and IgG2b antibodies were positively correlated to the IFN-γ secretion (R = 0.7063; p= 0.022 and R = 0.8735; p= 0.001, respectively) and negatively correlated to the parasite load in liver (R = −0.6560; p= 0.039 and R = −0.6963; p= 0.025, respectively). The anti-lysate IgG2b antibodies were also negatively correlated to the liver/ body relative weight (R = −0.6570; p= 0.039). Although the anti-NH36 or anti-*L.(L.) chagasi* lysate IgG2b antibodies are not protective by themselves, their increased levels serve as an indicator of a TH1 response, hence acting as a surrogate marker for natural resistance to VL [31]. The definition of an antigen that would discriminate antibodies generated during VL infection from antibodies induced by vaccination or natural resistance has been the object of intense investigation, mainly in the canine model of VL [30,32]. The complex promastigote lysate or soluble antigen of *L. (L.) infantum*[33] or *L. (L.) chagasi*[34] did not discriminate between infected and vaccinated dogs. On the other hand Rafati *et al*., [33] showed that higher IgG2 than IgG1 titres were detected against the recombinant antigens but not against the *L. (L.) infantum* lysate. For these reasons, in this investigation we compared the reactivity to the *L.(L.) chagasi* promastigote lysate and to the NH36 Nucleoside hydrolase protein, which is a phylogenetic conserved marker of the *Leishmania* genus [35], a very important enzyme in the DNA metabolism and establishment of the parasite infection [36] and an antigenic marker of the canine patent disease [37]. Both antigens discriminated between the C57 and KOB2 groups regarding their IgG2b and only the lysate allowed detection differences in IgG1. The IgG2b antibodies are expected to be increased in mice that show either vaccine induced protection or natural resistance to *Leishmania* infection [27].

**Figure 5:**
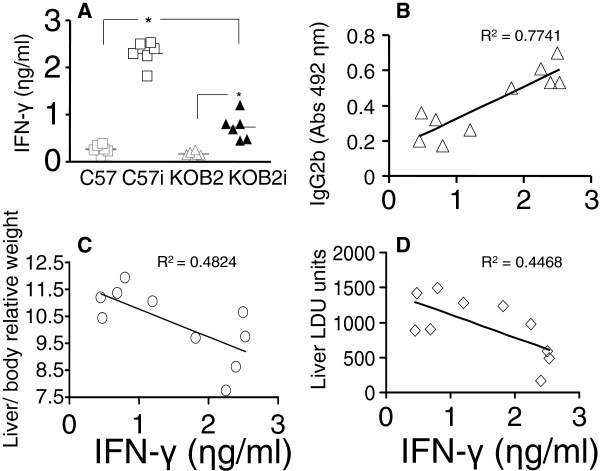
**Assessment of the IFN-γ cytokine secretion by splenocytes. **IFN-γ was assayed in the supernatants of splenocytes of C57 and KOB2 infected mice and in uninfected C57 and KOB2 control mice after 5 days of *in vitro* culture with the freeze and thawed lysate of *Leishmania (L.) chagasi* promastigotes. Data correspond to individual results of one experiment with 6 mice of each group obtained after sacrifice. In graph A, horizontal full lines represent the mean values of C57 normal uninfected (C57), C57 infected (C57i), KOB2 normal uninfected (KOB2) and KOB2 infected (KOB2i) mice. *p<0.05 indicates significant differences to the saline control as disclosed by the Kruskall Wallis (KW) and Mann Whitney (MW) non-parametrical tests. The results were expressed on graphs as scattering of individual values. R squared (R^2^) (coefficients of determination) estimates are shown on graphs. Trend lines were added.

Furthermore, the levels of IFN-γ secreted into the supernatants of splenocytes were significantly lower (p = 0.002) in the KOB2 infected mice (mean = 0.74 ηg/ml) than in the C57 infected mice (mean= 2.3 ηg/ml) (Figure 5). Both groups were different from their respective uninfected controls (p = 0.002 for each group) and neither the uninfected C57 or BOK2 mice secreted IFN-γ (p = 0.093). As shown for the DTH and IgG2b response, the IFN-γ in splenocyte supernatants was also a strong correlate for resistance to VL since its secretion was negatively correlated to the increase in liver LDU values (R = −0.6684; p= 0.035) and liver/ body relative weight (R = −0.6946; p= 0.026) and positively correlated to the IgG2b enhancement (R = 0.8817; p = 0.001). In the C57 model of resistance to VL therefore, the IFN-γ levels are also an indicator of a TH1 response to *Leishmania (L.) chagasi,* which is diminished in mice lacking the B2 receptor for bradykinin.

It might be argued that the quantification of the parasite load in bone marrow and spleen could also give a view of the influence of the bradykinin B2-receptor. Cotterell *et al*., [38] measured the parasite load in bone marrow of Balb/c mice infected with 2 x 10^7^ amastigotes of *L. (L.) donovani,* through limiting dilution analysis or tissues smears and about 100 amastigotes were recorded/1000 bone marrow cell nuclei at day 28 after infection. The bone marrow parasite load was also studied by limiting dilution in Balb/c mice infected with *L. (L.) infantum* by Carrión *et al*., [39]. Limiting dilution differs from the direct microscopical counting of parasites in smears, in that the limiting dilution technique involves the culture amplification of the true number of parasites of the organ. In spite of this amplification, only 10 parasites were detected in bone marrow while 100,000 were detected in liver [39], showing that the sensitivity for parasite detection in bone marrow is very low, and any significant difference between the parasite load of C57 and KOB2 mice in this organ are not expected to be detected.

On the other hand, regarding the evaluation of the spleen parasite load in our model, we observed a mean average ± SD of 91.11 ± 29.13 LDU units for C57BL/6 ^+/+^ mice and 70.43 ± 23.51 for C57BL/6 BR2 ^−/−^ mice, with no significant differences between the two groups. Similar levels (75 LDUs) were reported by Cotterell *et al*., [38] in Balb/c mice and much lower values were described by Ato *et al*., [23], who worked with C57BL/6 mice infected with a similar parasite inoculum (2 x 10^7^ amastigotes of *L.(L.) donovani*) and obtained an average of only 2 LDU at 14 days, and 10 LDU at 28 days after infection. This is probably due to the fact that in the classical model of mice VL acute infection after endovenous injection of the parasites, used by most authors [18,22,23,38,40], reviewed in [41] an early acute infection is detected in the liver and a later chronic infection in spleen, each one characterized by its own organ-specific immune responses [23,40]. We conclude that, in our model of acute VL infection, the best location to investigate the parasite load at day 30 after infection was the liver, where high parasite loads and significant differences between the C57BL/6 BR2^−/−^ and the C57BL/6 BR2^+/+^ wild type controls were found. Therefore, the liver gives the best view of the role of the BR2 receptor in resistance to VL at this earlier point of infection.

## Conclusions

In summary, our results suggest that the natural resistance to the development of VL in C57BL/6 mice is at least partially dependent of the presence and functionality of the kinin/B_2_R receptor pathway.

## Abbreviations

VL: Visceral Leishmaniasis; B2R: Bradykinin B2 Receptors; C57 and B2R ^**+/+**^: C57BL/6 mice; KOB2 and B2R ^**−/−**^: Bradykinin B2 receptors knock-out mice; DCs: Dendritic cells; DTH: Delayed type of hypersensitivity.

## Competing interests

The authors declare that there is no conflict of interest regarding the present work and the sponsors had no role in study design, data collection and analysis, decision to publish, or preparation of the manuscript.

## Authors’ contributions

DN carried out most of the experimental procedures. DFF and NM carried out part of the experimental procedures. AM, JS, and CBPS conceived the research. MP and CBPS contributed with data analysis and revision of the manuscript. CBPS wrote the manuscript. JS reviewed and edited the final MS. All authors read and approved the final version of the manuscript.
